# Supplementary figures and images for: Factors affecting spatio-temporal occurrence of sympatric civets in Parsa-Koshi Complex, Nepal
Source: PLoS One. 2025 Jun 11;20(6):e0325758. doi: 10.1371/journal.pone.0325758 (PMC12157121; doi:10.1371/journal.pone.0325758)

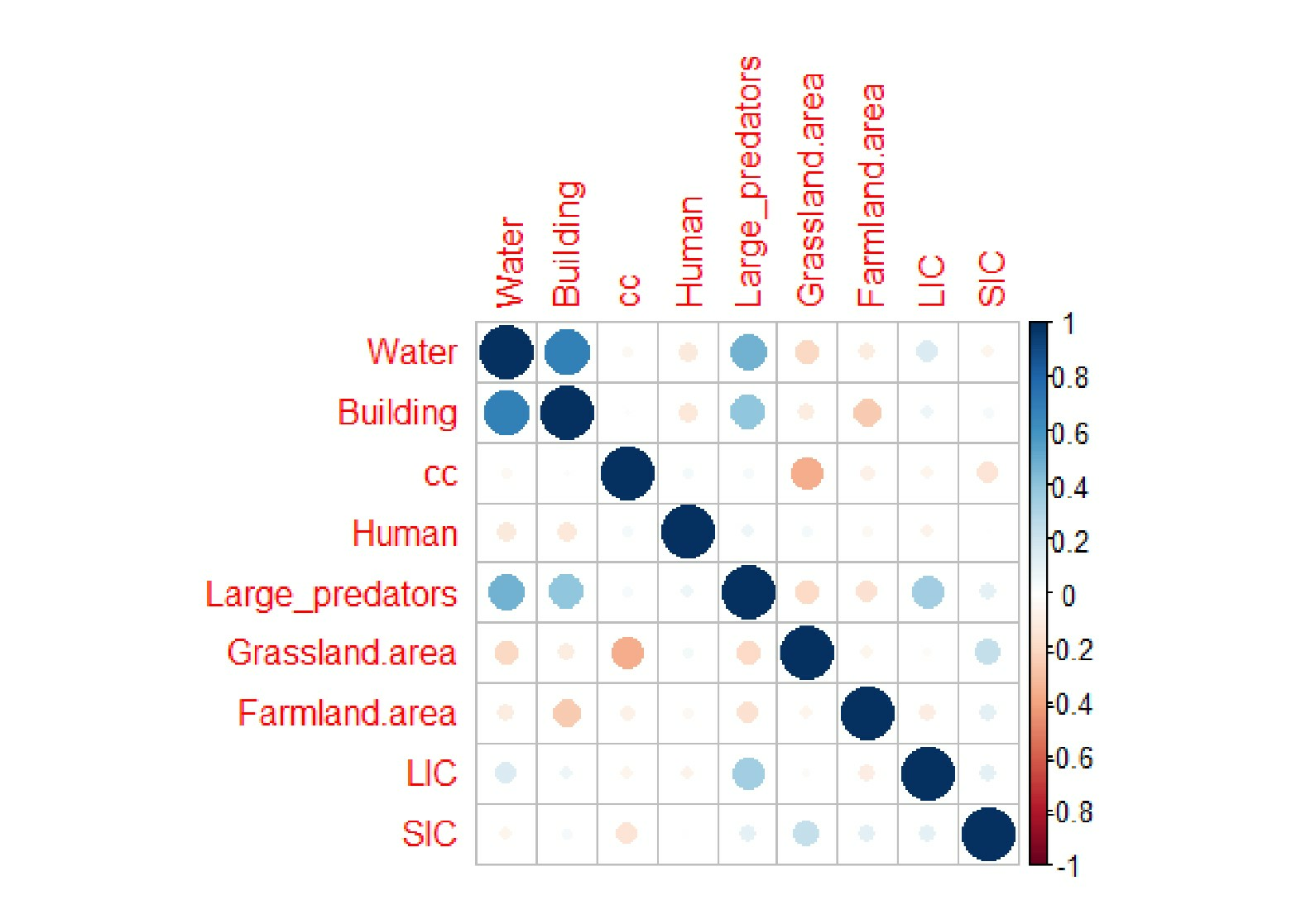

Supplement: S1 Fig — (TIF) [file pone.0325758.s001.tif]
